# Recognition of Autism Spectrum Disorder (ASD) symptoms and knowledge about some other aspects of ASD among final year medical students in Nigeria, Sub-Saharan Africa

**DOI:** 10.1186/s13104-015-1433-0

**Published:** 2015-09-18

**Authors:** M. O. Bakare, M. F. Tunde-Ayinmode, A. O. Adewuya, M. A. Bello-Mojeed, S. Sale, B. O. James, M. A. Yunusa, J. T. Obindo, M. N. Igwe, P. C. Odinka, C. J. Okafor, Y. O. Oshodi, K. M. Okonoda, K. M. Munir, A. O. Orovwigho

**Affiliations:** Federal Neuropsychiatric Hospital, Enugu, Enugu State Nigeria; Childhood Neuropsychiatric Disorders Initiatives (CNDI), Enugu, Enugu State Nigeria; College of Medicine, University of Ilorin, Ilorin, Kwara State Nigeria; College of Medicine, Lagos State University Ikeja, Ikeja, Lagos State Nigeria; Federal Neuropsychiatric Hospital, Yaba, Lagos State Nigeria; Faculty of Medicine, Bayero University Kano, Kano, Kano State Nigeria; Federal Neuropsychiatric Hospital, Uselu, Benin City,, Edo State Nigeria; College of Health Sciences, Usman Danfodio University Sokoto, Sokoto, Sokoto State Nigeria; Faculty of Medical Sciences, University of Jos, Jos, Plateau State Nigeria; College of Health Sciences, Ebonyi State University Abakaliki, Abakaliki, Ebonyi State Nigeria; College of Medicine, University of Nigeria Enugu Campus, Nsukka, Enugu State Nigeria; College of Medical Sciences, University of Calabar, Calabar, Cross River State Nigeria; College of Medicine, University of Lagos, Lagos, Lagos State Nigeria; Division of Developmental Medicine, Children’s Hospital and Harvard Medical School, University Center for Excellence in Developmental Disabilities (UCEDD), Boston, MA USA

**Keywords:** Knowledge, Symptoms, Autism Spectrum Disorder, Medical students, Education, Nigeria

## Abstract

**Background:**

Earlier studies suggest that knowledge about Autism Spectrum Disorder (ASD) among healthcare workers in Nigeria is low. This present study assessed the knowledge of Nigerian final year medical students about symptoms of ASD and some other aspects of ASD. This is a cross sectional descriptive study that drew a total of seven hundred and fifty-seven (757) final year medical students from ten (10) randomly selected fully accredited medical schools out of a total of twenty-seven (27) fully accredited medical schools in Nigeria. Sociodemographic and Knowledge about Childhood Autism among Health Workers (KCAHW) questionnaires were used to assess knowledge of final year medical students about ASD and obtain demographic information.

**Results:**

Only few, 218 (28.8 %) of the 757 final year medical students had seen and participated in evaluation and management of at least a child with ASD during their clinical postings in pediatrics and psychiatry. Knowledge and recognition of symptoms of ASD is observed to be better among this group of final year medical students as shown by higher mean scores in the four domains of KCAHW questionnaire. Knowledge about ASD varies across gender and regions. Misconceptions about ASD were also observed among the final year medical students.

**Conclusions:**

More focus needs to be given to ASD in the curriculum of Nigerian undergraduate medical students, especially during their psychiatry and pediatric clinical postings.

**Electronic supplementary material:**

The online version of this article (doi:10.1186/s13104-015-1433-0) contains supplementary material, which is available to authorized users.

## Background

There has been increased in Global prevalence of Autism Spectrum Disorder (ASD) over the last few decades [1, 2]. This increase prevalence is attributed in part to increase awareness 62 level about ASD [2].

While there has been increased awareness in parts of the world like Europe and United States, knowledge about ASD is still very low in most African countries [2–6]. Many of the healthcare workers in Sub-Saharan Africa, especially those in primary healthcare settings who are likely to first see children with neurodevelopmental disorders are unaware of symptoms of ASD [4]. This may prevent early diagnosis, which is essential in instituting early interventions. Thus, optimal prognosis of affected children may be compromised.

Medical students in the course of their study usually undergo posting in Pediatrics and Psychiatry which are the two specialties where they could learn about childhood neurodevelopmental disorders in general and specifically about ASD. According medical school curriculum in Nigeria, final year medical students would have gone through these two postings prior to their final Bachelor of Medicine; Bachelor of Surgery (M.B.B.S) examination. The medical school curriculum in Nigeria is regulated by Medical and Dental Council of Nigeria (MDCN) and is uniform across fully accredited Medical Schools, with mild variation of few days in clinical posting duration for special clinical postings like Psychiatry, Anesthesia, ENT among others.

These final year medical students in a couple of few months, would find themselves practicing as Interns at various levels of healthcare facilities like Primary, Secondary and Tertiary and may need to diagnose suspected cases of ASD, if they see one in the course of their practice. Lack of adequate knowledge about ASD among physicians has been associated with late diagnosis of ASD [7].

It is unclear what proportion of Nigerian final year medical students are exposed to evaluation and management of cases of ASD during the course of their training and recognize various symptoms and signs suggestive of diagnosis of ASD.

This study examined the knowledge of Nigerian final year medical students about various symptoms and signs of ASD and also their knowledge on some other aspects of ASD.

## Methods

**Fig. 1:**
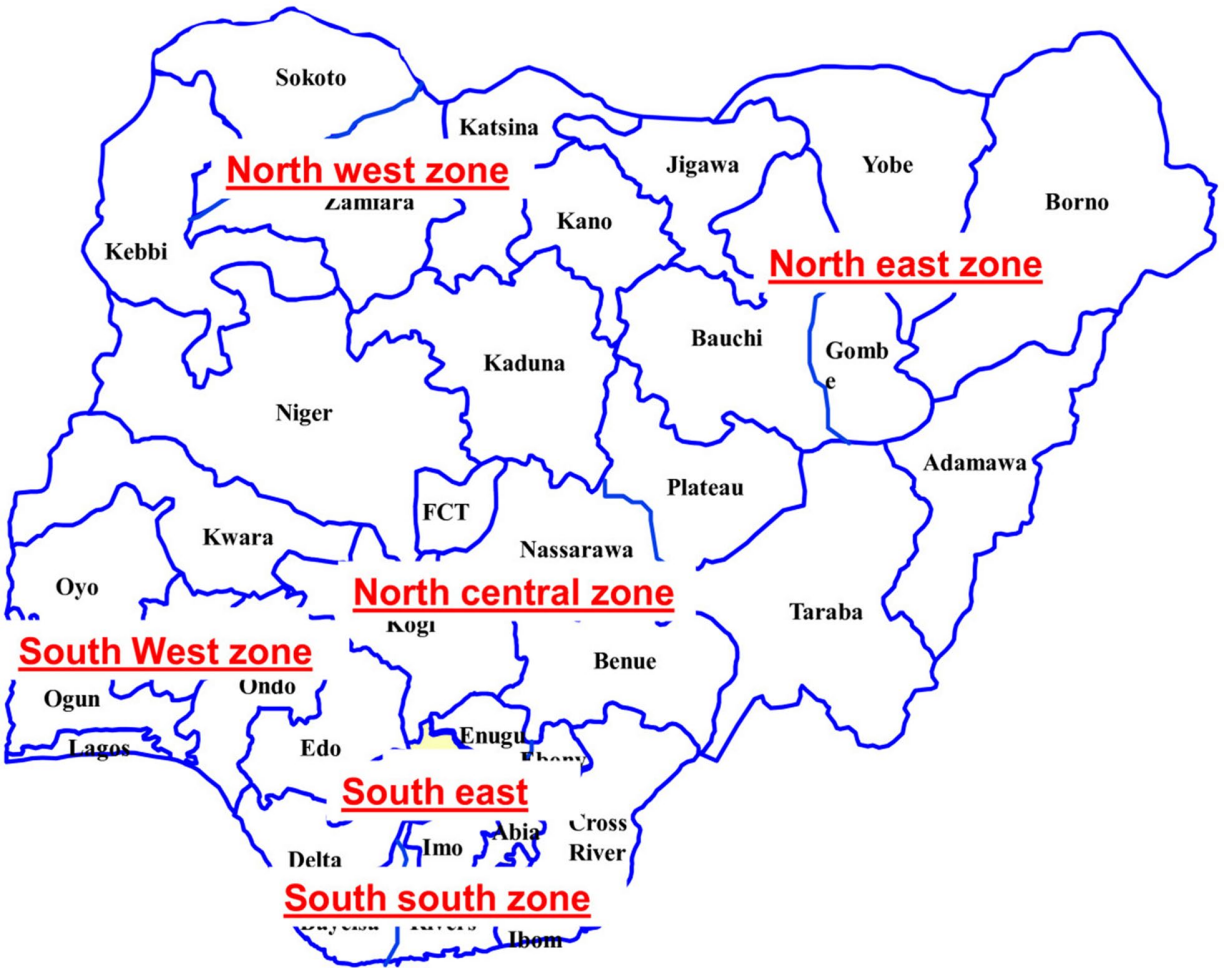
Nigerian map showing six geopolitical regions, thirty-six states and one FCT

The locations of the study are five out the six geopolitical zones of Nigeria. Nigeria, for ease of political and regional administration is divided into six geopolitical zones, thirty-six States and one Federal Capital Territory (FCT), which are: *North*-*East* (Yobe, Borno, Bauchi, Gombe, Adamawa and Taraba States); *North Central* (Niger, Nasarawa, Kwara, Kogi, Benue, Plateau and FCT); *North West* (Sokoto, Katsina, Jigawa, Kano, Zamfara, Kaduna and Kebbi States); *South East* (Anambra, Abia, Enugu, Ebonyi, and Imo States); *South*–*South* (Edo, Delta, Cross River, Akwa Ibom, Rivers and Bayelsa States); *South West* (Oyo, Osun, Ekiti, Ogun, Ondo and Lagos States). Figure 1 showed the Map of Nigeria with different geopolitical zones/regions of the country.

Five out of the six geopolitical zones of the country with a total of ten (10) medical schools, out of a total of twenty-seven (27) fully accredited medical schools in Nigeria [8] were involved in the study. Two fully accredited medical schools were randomly selected from each of the five geopolitical zones, making a total of ten fully accredited medical schools. The random selection of the ten medical schools led to nine States participating in the study.

Therefore, a total of ten (10) fully accredited medical schools selected from nine (9) States and five (5) geopolitical zones of Nigeria were involved in the study. The five geopolitical zones and nine (9) States involved in the study included: *North Central* (Kwara and Plateau States); *North West* (Kano and Sokoto States); *South East* (Ebonyi and Enugu States); *South*–*South* (Cross River and Edo States) and *South West* (Lagos State). The data from the *North East* region could not be included in the study because of the constraint experienced from school closure secondary to *‘Boko Haram’* insurgence. The data for the study was collected in the year 2012/2013 academic session across different geopolitical zones/regions of Nigeria.

The ten fully accredited medical schools that participated in the study included:

1. College of Medical Sciences, University of Calabar, Cross River State—*South*–*South.*
2. College of Health Sciences, Ebonyi State University Abakaliki, Ebonyi State—*SouthEast.*
3. College of Health Sciences, Igbinedion University Okada, Edo State—*South*–*South.*
4. College of Medicine, University of Nigeria Enugu Campus, Enugu State—*South East.*
5. Faculty of Medicine, Bayero University Kano, Kano State—*North West.*
6. College of Medicine, University of Ilorin, Kwara State—*North Central.*
7. College of Medicine, University of Lagos, Lagos State—*South West.*
8. College of Medicine, Lagos State University Ikeja, Lagos State—*South West.*
9. Faculty of Medical Sciences, University of Jos, Plateau State—*North Central.*
10. College of Health Sciences, Usman Danfodio University Sokoto, Sokoto State—*NorthWest.*

## Ethical consideration

The medical students were duly informed about the study and written informed consent was obtained from each of the students that participated in the study. The questionnaire did not contain any information that can identify the individual students. Ethical approval for this study was obtained from the Institutional Review Board (IRB) of Federal Neuropsychiatric Hospital, Enugu, Enugu State, Nigeria.

## Materials

### Sociodemographic questionnaire

This was used to obtain information such as age gender, marital status, religion, previous participation in evaluation and management of at least a child with ASD during pediatric and psychiatric postings and some other information.

*Knowledge about Childhood Autism among Health Workers (KCAHW) Questionnaire* This questionnaire had been described in our previous publications [3, 4]. The domains of KCAHW questionnaire are described below. Detail item questions and scoring of KCAHW questionnaire are included as Additional file 1: Appendixes S1 and S2 to this article. KCAHW questionnaire contained nineteen (19) item questions, which are divided into four (4) main domains.

*Domain 1* Contained eight (8) item questions that addressed the impairments in social interactions usually found in children with ASD.

*Domain 2* Contained only one (1) item question that addressed impairment in area of communication and language development found in ASD.

*Domain 3* Contained four (4) item questions that addressed area of obsession and compulsive pattern of behavior found in children with ASD.

*Domain 4* Contained six (6) item questions that addressed information on type of disorder ASD is, possible co-morbid conditions and usual age of onset of ASD.

Each of the correct answer on the 19 item questions is scored 1, so total scores of 8, 1, 4 and 6 are possible in each of the domains 1, 2, 3 and 4 respectively and a minimum score of zero (0) is possible in each of the domains [3].

### Procedure

This is a cross sectional descriptive study where total populations of final year medical students in each randomly selected fully accredited medical school were administered the questionnaires for the study. The Sociodemographic and KCAHW questionnaires were administered to final year medical students who have completed all their clinical postings in pediatrics and psychiatry in a classroom setting. The questionnaires were administered and collected back there and then. This is to prevent consultation of educational materials like textbooks or notebooks. Data were analyzed using Statistical Package for Social Science (SPSS), version16.

## Results

Seven hundred and fifty-seven (757) final year medical students drawn from ten (10) out of a total of twenty-seven (27) fully accredited medical schools across Nigeria were involved in the study [8]. The total population of female final year medical students was 300 (39.6 %) and 457 (60.4 %) for males final year medical students. The minimum and maximum ages were 19 and 42 years respectively. The mean age is 25.30 ± 2.90 year. The median and mode ages were both 25 years.

Table 1 showed the sex and age distribution of the final year medical students according to geopolitical regions.

**Table 1:** Age and gender distribution of the final year medical students according to geopolitical zones

Gender				Age			
Geopolitical zones	Male N (%)	Female N (%)	Total N (%)	Minimum age (years)	Maximum age (years)	Mean age (years)	Median age (years)
North Central	151 (69.6)	66 (30.4)	217 (28.7)	19	35	25.60 ± 2.47	25.00
North West	70 (56.9)	53 (43.1)	123 (16.3)	22	42	26.26 ± 2.64	26.00
South East	95 (60.9)	61 (39.1)	156 (20.6)	21	39	25.14 ± 2.80	25.00
South South	77 (55.8)	61 (44.2)	138 (18.2)	19	38	25.30 ± 75	24.00
South West	64 (52.0)	59 (48.0)	123 (16.3)	20	31	24.03 ± 2.43	24.00
Total	457 (60.4)	300 (39.6)	757 (100)	19	42	25.30 ± 2.90	25.00

### Previous participation in evaluation and management of at least a child with ASD during clinical postings in pediatrics and psychiatry

Out of a total of 757 final year medical students studied, 218 (28.8 %) had seen and participated in evaluation and management of at least a child with ASD during their clinical postings in pediatrics and psychiatry. The implication is that not all the medical students are privilege to see and participate in evaluation and management of children with ASD during their course of training.

Domain scores comparison between the group of students who had seen and participated in evaluation and management of at least a child with ASD and those who had not seen a case of ASD during the course of their clinical postings showed statistical significant difference in all the domains, with those who had participated in evaluation and management of at least a case of ASD more likely to have higher scores as shown in Table 2.

**Table 2:** Comparison of domain scores on KCAHW questionnaire of students with previous participation in evaluation and management of at least a case of ASD during their clinical postings and those without

Domain/Experience	N (%)	Mean scores	SD	*T* test/effect size
Domain 1				
Yes	218 (28.8)	6.5	1.6	t = 2.91; df = 755; *p* = 0.004
No	539 (71.2)	6.1	1.9	Cohen’s *d* = 0.24
Domain 2				
Yes	218 (28.8)	0.8	0.4	t = 2.71; df = 755; *p* = 0.007
No	539 (71.2)	0.7	0.5	Cohen’s *d* = 0.23
Domain 3				
Yes	218 (28.8)	2.9	1.1	t = 3.92; df = 755; *p* = 0.000
No	539 (71.2)	2.6	1.2	Cohen’s *d* = 0.32
Domain 4				
Yes	218 (28.8)	4.1	1.3	t = 4.92; df = 755; *p* = 0.000
No	539 (71.2)	3.6	1.4	Cohen’s *d* = 0.35

Yes**—**Previous participation in evaluation and management of at least a case of ASD

No—No previous participation in evaluation and management of a case of ASD

### Analysis of nineteen (19) item questions on KCAHW questionnaire

Item questions in domains 1–3 of KCAHW questionnaire provided information on recognition of symptoms of ASD among the final year medical students studied, while item questions in domain 4 provided information on their knowledge about some other aspects of ASD.

KCAHW questionnaire opens with an introductory statement as contained below:

The following behaviors best describe a child with Autism Spectrum Disorder (ASD):

1. Domain 1, Question 1 (D1Q1).

*Marked impairment in use of multiple non*-*verbal behaviors such as eye to eye contact, facial expression, body postures and gestures during social interaction*—599 (79.1 %) of a total of 757 (100.0 %) final year medical students admitted that this symptom is part of ASD symptoms, while 158 (20.9 %) do not agree or simply do not know.

2. Domain 1, Question 2 (D1Q2).

*Failure to develop peer relationship appropriate for developmental age*—693 (91.5 %) of a total of 757 (100.0 %) final year medical students admitted that this symptom is part of ASD symptoms, while 64 (8.5 %) do not agree or simply do not know.

3. Domain 1, Question 3 (D1Q3).

*Lack of spontaneous will to share enjoyment, interest or activities with other people*—657 (86.8 %) of a total of 757 (100.0 %) final year medical students admitted that this symptom constitute part of ASD symptoms, while 100 (13.2 %) do not agree or simply do not know.

4. Domain 1, Question 4 (D1Q4).

*Lack of social or emotional reciprocity*—616 (81.4 %) of a total of 757 (100.0 %) final year medical students admitted that this symptom constitute part of ASD symptoms, while 141 (18.6 %) do not agree or simply do not know.

5. Domain 1, Question 5 (D1Q5)*.

*Staring into open space and not focusing on anything specific*—511 (67.5 %) of a total of 757 (100.0 %) final year medical students admitted that this symptom could be part of ASD symptoms, while 246 (32.5 %) do not agree or simply do not know.

6. Domain 1, Question 6 (D1Q6).

*The child can appear as if deaf or dumb*—629 (83.1 %) of a total of 757 (100.0 %) final year medical students admitted that this could happen in children with ASD, while 215 (16.9 %) do not agree or simply do not know.

7. Domain 1, Question 7 (D1Q7).

*Loss of interest in the environment and surroundings*—596 (78.7 %) of a total of 757 (100.0 %) final year medical students admitted that this could be a symptom in children with ASD, while 161 (21.3 %) do not agree or simply do not know.

8. Domain 1, Question 8 (D1Q8)**.

*Social smile is usually absent in a child with Autism*—412 (54.4 %) of a total of 757 (100.0 %) final year medical students admitted that this could be a symptom in children with ASD, while 345 (45.6 %) do not agree or simply do not know.

9. Domain 2 (D2).

*Delay or total lack of development of spoken language*—542 (71.6 %) of a total of 757 (100.0 %) final year medical students agreed that this could be a symptom in children with ASD, while 215 (28.4 %) do not agree or simply do not know.

10. Domain 3, Question 1 (D3Q1).

*Stereotyped and repetitive movement (e.g. Hand or finger flapping or twisting)*—557 (73.6 %) of a total of 757 (100.0 %) final year medical students agreed that this could be part of symptoms in children with ASD, while 200 (23.4 %) do not agree or simply do not know.

11. Domain 3, Question 2 (D3Q2)***.

*May be associated with abnormal eating habit*—377 (49.8 %) of a total of 757 (100.0 %) final year medical students agreed that this could be part of symptoms in children with ASD, while 380 (50.2 %) do not agree or simply do not know.

12. Domain 3, Question 3 (D3Q3).

*Persistent preoccupation with parts of objects*—584 (77.1 %) of a total of 757 (100.0 %) final year medical students agreed that this could be part of symptoms in children with ASD, while 173 (22.9 %) do not agree or simply do not know.

13. Domain 3, Question 4 (D3Q4)*.

*Love for regimented routine activities*—502 (66.3 %) of a total of 757 (100.0 %) final year medical students recognized that this could be part of the symptoms in children with ASD, while 255 (33.7 %) do not.

14. Domain 4, Question 1 (D4Q1).

*Autism is Childhood Schizophrenia*—361 (47.7 %) of a total of 757 (100.0 %) final year medical students had misperception about ASD and childhood schizophrenia, while 396 (52.3 %) did not confuse ASD with childhood schizophrenia.

15. Domain 4, Question 2 (D4Q2).

*Autism is an auto*-*immune condition*—526 (69.5 %) of a total of 757 (100.0 %) final year medical students admitted that ASD is not an auto-immune disorder, while 231 (30.5 %) thought that ASD is an autoimmune disorder.

16. Domain 4, Question 3 (D4Q3).

*Autism is a neurodevelopmental disorder*—616 (81.4 %) of a total of 757 (100.0 %) final year medical students recognized that ASD is a neurodevelopmental disorder, while 141 (18.6 %) do not know this fact.

17. Domain 4, Question 4 (D4Q4).

*Autism could be associated with Mental Retardation*—604 (79.8 %) of a total of 757 (100.0 %) final year medical students agreed that ASD could be associated mental retardation or intellectual disability, while 153 (20.2 %) do not agree or unsure.

18. Domain 4, Question 5 (D4Q5).

*Autism could be associated with Epilepsy*—305 (40.3 %) of a total of 757 (100.0 %) final year medical students recognized that ASD could be associated with Epilepsy, while 452 (59.7 %) do not know or are unsure.

19. Domain 4, Question 6 (D4Q6).

*Onset of Autism is usually in*—404 (53.4 %) of a total of 757 (100.0 %) final year medical students admitted that onset of symptoms of ASD is usually in childhood, while 353 (46.6 %) opined that it is usually in neonatal or infancy stage.

### Proportion of final year medical students recognizing symptoms of ASD on item questions of KCAHW questionnaire

This study presumed that less than 70 % of the population of the final year medical students recognizing a particular symptom of ASD fall short of an acceptable expectation, the following item questions on KCAHW questionnaire revealed knowledge deficiency among the final year medical students using this criterion:

### Less than fifty percent of the population recognizing the symptoms***

This affected item question 11 (Domain 3, Question 2)—Less than 50 % of the final year medical students recognized that ASD symptoms could include abnormal eating habit as part of obsession and compulsive repetitive pattern of behavior or interest found in children with ASD.

### Less than sixty percent of the population recognizing the symptoms**

This affected item questions 8 (Domain 1, Question 8)—Less than 60 % of the final year medical students recognized that absence of social smile could be part of the symptoms of ASD in affected children.

### Less than seventy percent of the population recognizing the symptoms*

This affected item questions 5 (Domain 1, Question 5) and 13 (Domain 3, Question 4).

Item question 5 (Domain 1, Question 5)—Less than 70 % of the final year medical students recognized that staring into open space and not focusing on anything specific could be part of the symptoms of ASD in affected children.

Item question 13 (Domain 3, Question 4)—Less than 70 % of the final year medical students recognized that love for regimented routine activities could be part of symptoms of ASD in affected children.

So, major deficiency in knowledge are found on item questions 5 (Domain 1, Question 5); 8 (Domain 1, Question 8); 11 (Domain 3, Question 2); 13 (Domain 3, Question 4). This implied that the final year medical students in this study were not familiar with ‘abnormal eating habit’, ‘absence of social smile’, ‘non-specific gaze focus’ and ‘love for regimented routine activities’ as possible symptoms of ASD.

### Mean scores in different domains of KCAHW questionnaire according to gender and geopolitical regions

As earlier highlighted about KCAHW Questionnaire;

*Domain 1* Represents ASD symptoms related to *Impairment in Social Interaction.*

*Domain 2* Represents ASD symptoms related to *Impairment in Communication and Language Development.*

*Domain 3* Represents ASD symptoms related to *Repetitive, Obsessive and Compulsive pattern of behavior.*

*Domain 4* Represents information related to *co*-*morbidity and other aspects of knowledge about ASD.*

The pattern of mean scores distribution in the four domains of KCAHW questionnaire in respect to Gender and Regions are shown in Tables 3 and 4 respectively.

**Table 3:** Gender and mean scores in the four domains of KCAHW questionnaire

Gender	N (%)	Domain 1	Domain 2	Domain 3	Domain 4
Male	457 (60.4)	6.2 ± 1.9	0.7 ± 0.4	2.7 ± 1.3	3.9 ± 1.4
Female	300 (39.6)	6.2 ± 1.7	0.7 ± 0.5	2.7 ± 1.1	3.6 ± 1.4
Total	757 (100)	6.2 ± 1.8	0.7 ± 0.5	2.7 ± 1.2	3.8 ± 1.4
Statistics	(T-test)	*p* = 0.968	*p* = 0.676	*p* = 0.761	*p* = 0.011

Maximum scores of 8, 1, 4 and 6 are possible in domains 1, 2, 3 and 4 respectively. A minimum score of 0 is possible in any of the four domains

There is no statistical significant difference in the mean scores across genders in domains 1, 2 and 3, but domain 4 showed statistical significant difference with male gender performing better in this domain

**Table 4:** Regions and mean scores in the four domains of KCAHW questionnaire

Gender	N (%)	Domain 1	Domain 2	Domain 3	Domain 4
North Central	217 (28.7)	6.6 ± 1.5	0.8 ± 0.4	2.8 ± 1.1	4.0 ± 1.3
North West	123 (16.3)	6.5 ± 1.7	0.8 ± 0.4	2.5 ± 1.3	4.1 ± 1.4
South East	156 (20.6)	6.4 ± 1.6	0.7 ± 0.5	2.9 ± 1.0	3.5 ± 1.4
South–South	138 (18.2)	5.6 ± 2.1	0.5 ± 0.5	2.2 ± 1.3	3.4 ± 1.4
South West	123 (16.3)	5.8 ± 2.2	0.7 ± 0.5	2.7 ± 1.2	3.8 ± 1.3
Total	757 (100)	6.2 ± 1.8	0.7 ± 0.5	2.7 ± 1.2	3.8 ± 1.4
Statistics	(ANOVA)	F = 9.22 *p* = 0.000	F = 10.20 *p* = 0.000	F = 8.82 *p* = 0.000	F = 9.33 *p* = 0.000

Maximum scores of 8, 1, 4 and 6 are possible in domains 1, 2, 3 and 4 respectively. A minimum score of 0 is possible in any of the four domains

The four domains of KCAHW questionnaire in relation to the Regions showed statistical significant difference in the mean scores, with North Central, North Central, South-East and North West having the highest mean scores in domains 1, 2, 3 and 4 respectively

## Discussion

Few medical students, less than thirty percent of the total population had seen and participated in evaluation and management of at least a case of ASD. Knowledge and recognition of symptoms of ASD is observed to be better among the group of final year medical students with previous experience of participation in evaluation and management of cases of ASD.

Major deficiency in recognition of ASD symptoms were observed in item questions 5, 8, 11 and 13, which are item questions in domain 1 and 3 of KCAHW questionnaire. Final year medical students in this study were not familiar with abnormal eating habit, absence of social smile, non-specific gaze focus and love for regimented routine activities as symptoms of ASD.

Substantial proportion of the final year medical students confused ASD with childhood schizophrenia. Medical undergraduate curriculum in this environment needs to focus on correcting this misperception.

Though, previous studies had documented association between ASD and auto-immune conditions [9–11], there is yet no conclusive scientific evidence to suggest that ASD is an autoimmune condition. About a third of the final year medical students had a misconception about ASD being an auto-immune condition.

Majority of the final year medical students, over eighty percent recognized that ASD is a neurodevelopmental disorder. Majority of the students also recognized that ASD is likely to be associated with mental retardation or intellectual disability. Previous studies had shown that diagnosis of ASD in Africa is rarely made exclusively of intellectual disability [12, 13].

Less than half of the population of the medical students recognized that ASD may be associated with Epilepsy. Epilepsy has been reported as a common co-morbidity with ASD among African children [13].

Though, symptoms of ASD are now recognizable during infancy because of various methods of early screening [14], the usual manifestation of ASD symptoms is in childhood. A little above half of the final year medical students admitted that the usual onset of ASD symptoms is in childhood.

Knowledge about ASD among the final year medical students also varies across gender and geopolitical regions as reflected in Tables 3 and 4. For example, there is no statistical significant difference in the mean scores across genders in domains 1, 2 and 3, but domain 4 showed statistical significant difference with male gender performing better in this domain. The four domains of KCAHW questionnaire in relation to the Regions also showed statistical significant difference in the mean scores, with North Central, North Central, South-East and North West having the highest mean scores in domains 1, 2, 3 and 4 respectively.

### Limitations

The main limitation of this cross sectional survey is that one of the six geopolitical regions in the country is not represented. The data from North-East region could not be included in the study because of constraint experienced from school closure secondary to *“Boko Haram”* insurgency during the period of data collection. The data could have been more robust if it were possible to involve every region of the country.

Another limitation is that there is paucity of data in African sub-region on this topic and very few studies are available to compare directly the findings of this study.

Despite the limitations, we believe that the salient findings of this study and the conclusions resulting from the findings are still germane.

## Conclusions

In view of the observation that knowledge and recognition of symptoms of ASD is better among the group of final year medical students with previous experience seeing and participating in evaluation and management of cases of ASD, audio-visual teaching aids showing various symptoms of ASD, evaluation and management might be useful in improving the recognition of ASD symptoms among Nigerian medical students. Many medical students may not have the opportunity of seeing real cases of ASD because of infrequent presentation of these cases to orthodox medical care in Sub-Saharan Africa, largely because of associated societal stigma and abnormal help seeking behavior [15, 16]. The finding of this present study showing that less than thirty percent of the medical students’ population had actually seen at least a case of ASD and participated in evaluation and management, further reinforce this observation.

More focus need to be given to ASD and other neurodevelopmental disorders in the curriculum of undergraduate medical students in Nigeria and other Sub-Saharan Africa countries, especially during their psychiatry and pediatric clinical postings.

## Additional file

**Additional file 1.** Knowledge about Childhood Autism among Health Workers (KCAHW) questionnaire.

## Abbreviations

ENT: Ear, nose and throat
ASD: Autism Spectrum Disorder

## References

[1] MMWR. Prevalence of Autism Spectrum Disorders—autism and disabilities monitoring network, 14 sites, United States. 2008;61:SS-3. http://www.cdc.gov/mmwr/pdf/ss/ss6103.pdf. 22456193

[2] Elsabbagh M, Divan G, Koh YJ, Kim YS, Kauchali S, Marcín C, Montiel-Nava C, Patel V, Paula CS, Wang C, Yasamy MT, Fombonne E (2012). Global prevalence of autism and other pervasive developmental disorders. Autism Res.

[3] Bakare MO, Ebigbo PO, Agomoh AO, Menkiti NC. Knowledge about childhood autism among health workers (KCAHW) questionnaire: description, reliability and internal consistency; Clinical practice and epidemiology in mental health. 2008;4:17. www.cpementalhealth.com/content/4/1/17. 10.1186/1745-0179-4-17 PMC2430959 18538020

[4] Bakare MO, Ebigbo PO, Agomoh AO, Eaton J, Onwukwe JU, Onyeama GM, Okonkwo KO, Igwe MN, Orovighwo AO, Aguocha CM. Knowledge about childhood autism and opinion among healthcare workers on availability of facilities and law caring for the needs and rights of children with childhood autism and other developmental disorders in Nigeria. BMC Pediatr. 2009,9:12. 10.1186/1471-2431-9-12 PMC2650693 19216754

[5] Igwe MN, Bakare MO, Onyeama GM, Okonkwo KO. Factors influencing knowledge about childhood autism among final year undergraduate medical, nursing and psychology students in University of Nigeria, Enugu, Nigeria. Ital J Pediatr. 2010;36:37. http://www.ijponline.net/content/36/1/44 10.1186/1824-7288-36-44 PMC2894024 20540799

[6] Igwe MN, Ahanotu AC, Bakare MO, Achor JU, Igwe C. Assessment of knowledge about childhood autism among paediatric and psychiatric nurses in Ebonyi State, Nigeria. Child Adoles Psychiatry Ment Health. 2011;5:1. http://www.capmh.com/content/5/1/1 10.1186/1753-2000-5-1 PMC3022827 21214953

[7] Rhoades RA, Scarpa A, Salley B (2007). The importance of physician knowledge of autism spectrum disorder: results of a parent survey. BMC Pediatr.

[8] Medical and Dental Council of Nigeria (MDCN). Fully accredited medical/dental schools in Nigeria. 2014;www.mdcnigeria.org. Accessed on 22nd April, 2014.

[9] Ashwood P, Van de Water J (2004). Is autism an autoimmune disease?. Autoimmun Rev.

[10] Enstrom AM, Van de Water JA, Ashwood P (2009). Autoimmunity in autism. Curr Opin Investig Drugs.

[11] Careaga M, Van de Water J, Ashwood P. Immune dysfunction in autism: a pathway to treatment. Neurotherapeutics. 2010;7(3):283–92. 10.1016/j.nurt.2010.05.003 PMC5084232 20643381

[12] Bakare MO, Munir KM (2011). Autism spectrum disorders (ASD) in Africa: a perspective. Afr J Psychiatry..

[13] Bakare MO, Munir KM. Autism spectrum disorders in Africa. In: Mohammadi M-R, editor. A comprehensive book on Autism Spectrum Disorders, chap 10. InTech; 2011. ISBN: 978-953-307-494-8. http://www.intechopen.com/books/a-comprehensive-book-on-autism-spectrum-disorders/autism-spectrum-disorders-in-africa.

[14] Yoon JM, Vouloumanos A (2014). When and how does autism begin?. Trends Cogn Sci..

[15] Bakare MO, Munir KM (2011). Excess of non-verbal cases of autism spectrum disorders presenting to orthodox practice in Africa—a trend possibly resulting from late diagnosis and intervention. S Afr J Psychiatry.

[16] Bello-Mojeed MA, Bakare MO, Munir K. Identification of Autism Spectrum Disorders (ASD) in Africa: Need for shifting research and public health focus. 2013. http://www.springrreference.com/index/chapterdbid/331260

