# Supplementary material for: Recognition of Autism Spectrum Disorder (ASD) symptoms and knowledge about some other aspects of ASD among final year medical students in Nigeria, Sub-Saharan Africa
Source: BMC Res Notes. 2015 Sep 18;8:454. doi: 10.1186/s13104-015-1433-0 (PMC4574575; doi:10.1186/s13104-015-1433-0)
Supplement: Supplementary file 1 — Additional file 1. Knowledge about Childhood Autism among Health Workers (KCAHW) questionnaire. [file 13104_2015_1433_MOESM1_ESM.doc]

**Appendix 1**

***Knowledge about Childhood Autism among Health Workers (KCAHW) Questionnaire***

**Please do not consult formal text books to answer these questions**. **Thank you for your time**.

The following behaviors best describe a child with Autism Spectrum Disorders (ASD)

**Domain 1**

i. Marked impairment in use of multiple non-verbal behaviors such as eye to eye contact, facial expression, body postures and gestures during social interaction? (A) Don't Know, (B) Yes, (C) No

ii. Failure to develop peer relationship appropriate for developmental age? (A) Don't Know, (B) Yes, (C) No

iii. Lack of spontaneous will to share enjoyment, interest or activities with other people? (A) Don't Know, (B) Yes, (C) No

iv. Lack of social or emotional reciprocity? (A) Don't Know, (B) Yes, (C) No

v. Staring into open space and not focusing on anything specific? (A) Don't Know, (B) Yes, (C) No

vi. The child can appear as if deaf or dumb? (A) Don't Know, (B) Yes, (C) No

vii. Loss of interest in the environment and surroundings? (A) Don't Know, (B) Yes, (C) No

viii. Social smile is usually absent in a child with Autism? (A) Don't Know, (B) Yes (C) No

**Domain 2**

i. Delay or total lack of development of spoken language? (A) Don't Know (B) Yes (C) No

**Domain 3**

i. Stereotyped and repetitive movement (e.g. Hand or finger flapping or twisting)? (A) Don't Know (B) Yes, (C) No

ii. May be associated with abnormal eating habit? (A) Don't Know, (B) Yes, (C) No

iii. Persistent preoccupation with parts of objects? (A) Don't Know, (B) Yes, (C) No

iv. Love for regimented routine activities? (A) Don't Know, (B) Yes, (C) No

**Domain 4**

i. Autism is Childhood Schizophrenia? (A) Don't Know, (B) Yes, (C) No

ii. Autism is an auto-immune condition? (A) Don't Know, (B) Yes, (C) No

iii. Autism is a neuro-developmental disorder? (A) Don't Know, (B) Yes, (C) No

iv. Autism could be associated with Mental Retardation? (A) Don't Know, (B) Yes, (C) No

v. Autism could be associated with Epilepsy? (A) Don't Know, (B) Yes, (C) No

vi. Onset of Autism is usually in, (A) Neonatal age, (B) Infancy, (C) Childhood

**Appendix 2**

***Scoring of Knowledge about Childhood Autism among Health Workers (KCAHW) Questionnaire***

**Domain 1**

i. Marked impairment in use of multiple non-verbal behaviors such as eye to eye contact, facial expression, body postures and gestures during social interaction? (A) 0, (B) 1, (C) 0

ii. Failure to develop peer relationship appropriate for developmental age? (A) 0, (B) 1, (C) 0

iii. Lack of spontaneous will to share enjoyment, interest or activities with other people? (A) 0, (B) 1, (C) 0

iv. Lack of social or emotional reciprocity? (A) 0, (B) 1, (C) 0

v. Staring into open space and not focusing on anything specific? (A) 0, (B) 1, (C) 0

vi. The child can appear as if deaf or dumb? (A) 0, (B) 1, (C) 0

vii. Loss of interest in the environment and surroundings? (A) 0, (B) 1, (C) 0

viii. Social smile is usually absent in a child with Autism? (A) 0, (B) 1 (C) 0

**Domain 2**

i. Delay or total lack of development of spoken language? (A) 0 (B) 1 (C) 0

**Domain 3**

i. Stereotyped and repetitive movement (e.g. Hand or finger flapping or twisting)? (A) 0, (B) 1, (C) 0

ii. May be associated with abnormal eating habit? (A) 0, (B) 1, (C) 0

iii. Persistent preoccupation with parts of objects? (A) 0, (B) 1, (C) 0

iv. Love for regimented routine activities? (A) 0, (B) 1, (C) 0

**Domain 4**

i. Autism is Childhood Schizophrenia? (A) 0, (B) 0, (C) 1

ii. Autism is an auto-immune condition? (A) 0, (B) 0, (C) 1

iii. Autism is a neuro-developmental disorder? (A) 0, (B) 1, (C) 0

iv. Autism could be associated with Mental Retardation? (A) 0, (B) 1, (C) 0

v. Autism could be associated with Epilepsy? (A) 0, (B) 1, (C) 0

vi. Onset of Autism is usually in, (A) 0, (B) 0, (C) 1
